# Bispecific CD3-HAC carried by E1A-engineered mesenchymal stromal cells against metastatic breast cancer by blocking PD-L1 and activating T cells

**DOI:** 10.1186/s13045-019-0723-8

**Published:** 2019-04-25

**Authors:** Yuanyuan Yang, Xiaolong Zhang, Fangzhen Lin, Mengshang Xiong, Dongmei Fan, Xiangfei Yuan, Yang Lu, Yuewen Song, Yizi Zhang, Mu Hao, Zhou Ye, Yanjun Zhang, Jianxiang Wang, Dongsheng Xiong

**Affiliations:** 1 State Key Laboratory of Experimental HematologyInstitute of Hematology and Hospital of Blood Diseases, Chinese Academy of Medical Science and Peking Union Medical College, Tianjin, 300020 People’s Republic of China; 2 Department of Pharmacy, National Clinical Research Center of Cancer, Key Laboratory of Cancer Prevention and Therapy, Tianjin’s Clinical Research Center for Cancer, Tianjin Medical University Cancer Institute and Hospital, Tianjin, 300060 People’s Republic of China; 3 Tianjin Institute of Integrative Medicine for Acute Abdominal Diseases, Tianjin Nankai Hospital, Tianjin, 300100 People’s Republic of China; 4 Central Hospital of Karamay, Karamay, Xinjiang 834000 People’s Republic of China

**Keywords:** PD-L1, CD3-HAC, MSC, Metastatic, Immunotherapy

## Abstract

**Background:**

PD-1/PD-L1 blockade can confer durable benefits in the treatment of metastatic cancers, but the response rate remains modest and potential adverse effects occur sometimes. Concentrating immunotherapeutic agents at the site of disease was believed to break local immune tolerance and reduce systemic toxicity. E1A-engineered mesenchymal stromal cell (MSC.E1A) was an attractive transfer system that preferentially homing and treating cancer metastasis, through which the tumor cells were modified by locally replicated adenoviruses to release CD3-HAC, a bifunctional fusion protein that anti-CD3 scfv linked with high-affinity consensus (HAC) PD-1. Subsequently, CD3-HAC, wbich was bound on PD-L1-positive breast cancer cells, recruited T cells to exhibit a potent antitumor immunity incombination with immune checkpoint blockade.

**Methods:**

We constructed the CD3-HAC gene driven by human telomerase reverse transcriptase (hTERT) promoter into an adenoviral vector and the E1A gene into the lentiviral vector. The homing property of MSCs in vivo was analyzed with firefly luciferase-labeled MSCs (MSC.Luc) by bioluminescent imaging (BLI). The cytotoxicity of T cells induced by CD3-HAC towards PD-L1-positive cells was detected in vitro and in vivo in combination with 5-FU.

**Results:**

Our data suggest that CD3-HAC could specifically bind to PD-L1-positive tumor cells and induce lymphocyte-mediated lysis effectively both in vitro and in vivo. The intervention with HAC diminished the effects of PD-1/PD-L1 axis on T cells exposed to MDA-MB-231 cells and increased lymphocytes activation. MSCs infected by AdCD3-HAC followed by LentiR.E1A could specially migrate to metastasis of breast cancer and produce adenoviruses in the tumor sites. Furthermore, treatment with MSC.CD3-HAC.E1A in combination with 5-FU significantly inhibited the tumor growth in mice.

**Conclusions:**

This adenovirus-loaded MSC.E1A system provides a promising strategy for the identification and elimination of metastasis with locally released immuno-modulator.

**Electronic supplementary material:**

The online version of this article (10.1186/s13045-019-0723-8) contains supplementary material, which is available to authorized users.

## Background

Breast cancer accounts for the most commonly diagnosed cancer and the leading cause of cancer death among females [1]. Triple-negative breast cancer (TNBC) was characterized by the lack of estrogen receptor (ER), progesterone receptor (PR), and HER2. Patients with TNBC had an increased likelihood of distant recurrence, and the median survival time from recurrence to death was only 9 months [2]. The clinical treatment for the disseminated cancer was limited by ineffective chemotherapy and unfeasible surgical resection [3].

Recently, immune checkpoint blockade targeting the programmed death-1 (PD-1)/programmed death-ligand 1 (PD-L1) pathway [4, 5] had induced remarkable clinical outcomes in various malignancies [6–10]. Furthermore, it was also a promising candidate to treat metastatic TNBC due to more lymphocytes infiltration and PD-L1 expression in the tumor microenvironment resulted from the higher genomic instability and mutational burden. However, single agent of PD-1 or PD-L1-blocking therapy hardly converted the “cold” tumors to be “hot” [11, 12], due to poor T cell priming or even immunological ignorance in deep immunosuppress tumor phenotype. For solving this problem, we constructed a bispecific fusion protein named CD3-HAC, which was constituted by anti-CD3 scfv and high-affinity consensus (HAC) PD-1. The HAC is a mutant of PD-1 ectodomain which shows superior therapeutic efficacy in mice compared with antibodies [13]. We hypothesized that bifunctional fusion protein CD3-HAC served as an effective mean to neutralize PD-L1-mediated immune suppression and simultaneously enhance insufficient T cell priming, bypassing the recognition of the MHC and impaired antigen-specific responses [14, 15].

It had been reported that intratumoral release of immune checkpoint inhibitors was a promising avenue to avoid the fatal side effects caused by the conventional intravenous infusion, such as myocarditis and diffuse melanosis cutis. Obviously, local immunotherapy could boost more effective antitumor immune response and reverse cancer immunotolerance, while leading less autoimmune toxicity [16]. Recently, ongoing clinical trials [16] and preclinical models [17–19] had shown enhanced antitumor outcome by in situ injection or using biomaterial vehicles. However, the use of intratumoral immunotherapy was limited to treat metastatic tumors because of the insufficient size for injection and the low resolution of current imaging techniques in detecting micrometastases. Therefore, a suitable delivery vehicle carrying drugs for metastatic tumors may conquer this trouble.

Mesenchymal stromal cells (MSCs), appealing cell-based vectors, could be employed to realize the local delivery of therapeutics. Systemically infused MSCs could selectively migrate towards malignant tumors and track microscopic metastases [20, 21]. Superior to the invasive and damaged forms of administration, MSCs were widely demonstrated safely [22, 23]. Additionally, genetically engineered MSCs retained these properties and had been served as a promising targeted system for tumor therapy [24–29]. In previous research, we had underscored the utility of E1A-modified MSCs (MSC.E1A) as transporters and amplifiers for adenovirus in the subcutaneous hepatic xenograft tumor model [30].

In this study, we hypothesized that adenovirus loading with expression frame of CD3-HAC fusion protein was delivered to the local site of metastasis by MSC.E1A and infected tumor cells. Subsequently, CD3-HAC were released locally and enhanced antitumor responses through specific lysis and PD-1/PD-L1 blockade (Additional file 1: Figure S1). We reasoned that it would be a promising approach to eliminate cancer, especially for distal residual lesions.

## Materials and methods

### Cell lines and cell culture

Human breast cancer cell lines (MDA-MB-231 and MCF-7), embryonic renal cell line 293 A (Institute of Hematology and Blood Diseases Hospital Chinese Academy of Medical Sciences and Peking Union Medical College, PUMC, Tianjin, China), and human embryonic kidney cell-derived 293T cell line (kindly provided by Professor Cheng Tao, PUMC) were maintained in DMEM (Gibco, 1791922) supplemented with 10% FBS.

### MSC preparation

MSCs were isolated from human umbilical cord Wharton’s jelly (WJ) as previously described [31]. MSCs were cultured at a density of 8 × 10^3^ cell/cm^2^ in DF-12 medium (Invitrogen, USA) supplemented with 2 mM L-glutamine and 10% FBS (Gibco, USA). When cells reached 80~90% confluence, they were detached using a 0.125% trypsin/1 mM EDTA solution and re-seeded using the same growth medium for subsequent passages. For all experiments, early-passage MSCs (3P to 5P) were used.

### Expression and purification of CD3-HAC

293T cells were transfected with pcDNA3.1(+)-CD3-HAC using Lipofectamine 2000 (Invitrogen, USA) according to the manufacturer’s protocol. After 48 h of transfection, supernatants were collected by centrifugation at 500×*g* for 10 min at 4 °C to clear cellular debris. The secretory CD3-HAC in the supernatants were purified by 6×His-tag affinity chromatography (GE Healthcare, Sweden) according to the manufacturer’s instruction. The purified preparations were quantified by Western blot analysis and used for cell-binding assays in vitro.

### CD3-HAC binding detection on transduced cells

To confirm the expression of CD3-HAC protein, Western blot analysis was performed. And the cell surface binding of CD3-HAC was determined by flow cytometry and immunofluorescence analysis. MDA-MB-231 cells or MCF-7 cells were infected with AdCD3-HAC, AdHAC, AdCD3scfv, or Adtrack at 100 MOI for 48 h, respectively. The following detections were performed as described previously [32].

### Cytotoxicity assays in vitro

MDA-MB-231 cells or MCF-7 cells were infected by AdCD3-HAC, AdCD3scfv, AdHAC, and Adtrack at 100 MOI for 48 h. Then, the adenovirus-loaded cells were seeded to 96-well plates (1 × 10^4^/well). The next day, peripheral blood mononuclear cells (PBMCs) pretreated with IL-2 for 72 h were added at different effector to target (E:T) cell ratios ranging from 20:1 to 2.5:1. After 10 h, the specific lysis of target cells was detected by LDH release assay according to the manufacturer’s instruction. The percentage of cell lysis was calculated as the following formula: Cytotoxicity = (Experimental − effector spontaneous − target spontaneous)/(target maximum − target spontaneous) × 100%. For the 5-FU-enhanced cytotoxicity assay, MDA-MB-231 cells were pretreated with or without 5-FU (0.25 μg/mL) for 24 h followed by adenovirus infection. Forty-eight hours later, target cells were plated to 96-well plates (1 × 10^4^/well), and PBMCs were added at E:T ratio of 10:1. The following processes were performed as described above.

### Restoration of lymphocyte activity with HAC

A MDA-MB-231 cell line constitutively expressing membrane-bound anti-CD3scfv, named 231.CD3, was established. For the first round stimulation, PBMCs were incubated with 231.CD3 cells at E:T ratio of 5:1 for 3 days. Then, the floating cells were harvested and washed twice by PBS. For the second round of co-incubation with 231.CD3 cells, the E:T ratio was turned to 1:5 and lasted for 5 days with or without HAC (33 pmol/mL). Finally, the secreted IFN-γ in supernatant was measured by ELISA.

### Real-time PCR

Total RNA was extracted from suspension cells using Trizol reagent (Invitrogen, USA) following the manufacturer’s protocol. The complementary DNA (cDNA) was generated using OligdT primers and M-MLV reverse transcriptase (Invitrogen, USA) with 2 μg total RNA. Real-time PCR was performed using QuantStudio 5 real-time PCR system (Applied Biosystems, USA), in combination with SYBR Green (Takara, Dalian, China). The primers were designed as follows: 5′-AAGTCAGCTCCACTGAAGCT-3′ and 5′-GGTAGGTTTGGTGGAAGGAG-3′ for perforin, 5′-GCTTATCTTATGATCTGGGATC-3′ and 5′-AAGTCAGATTCGCACTTTCGA-3′ for granzyme B. Relative transcript expression was normalized to that of GAPDH mRNA.

### Co-culture study and assessment of apoptosis

MDA-MB-231 cells loaded with AdHAC or Adtrack were co-cultured with Jurkat leukemia T cells at the ratio of 10:1 for 24 h. Before co-incubation, Jurkat cells were labeled with Cell Trace™ Far Red (Thermofisher) for flow cytometry analysis. The extent of apoptosis in Jurkat cells was determined by flow cytometry using FITC-Annexin V (BD) according to the manufacturer’s instruction.

### Package of replication-defective adenovirus in E1A-modified MSCs in vitro

MSCs were plated on 6-well plates (1 × 10^5^/well) and were infected with Adtrack at 500 MOI. On the next day, cells were washed twice with PBS and transduced with LentiR.E1A (lentiviral expression vector for E1A was shown in Additional file 1: Figure S2) with 8 μg/mL polybrene (Sigma). Co-infected MSCs and corresponding supernatants were harvested at indicated time, from which the adenoviral DNA was extracted using High Pure Viral Nucleic Acid Extraction Kit (Roche). Digital PCR was performed by QX200 Droplet Digital PCR system to examine the exact copy number of hexon gene (nucleotides 21049–21334 of Ad5), representing the quantity of viral particles. The primers were designed as follows: 5′-GGTGGCCATTACCTTTGACTCTTC-3′ and 5′-CCACCTGTTGGTAGTCCTTGTATTTAGTATCATC-3′. Finally, the DNA copy numbers were converted to total adenovirus particle counts by multiplying a certain dilution ratio.

### Breast cancer lung metastasis animal models

MDA-MB-231 (231) or Luc-MDA-MB-231 (231-Luc) breast cancer cells (1 × 10^6^ per mouse) were infused intravenously (i.v.) into BALB/c nude mice (female, 5–6 weeks of age; PUMC, China). All animal studies were performed according to the guidelines under the Animal Ethics Committee of Chinese Academy of Medical Sciences and Peking Union Medical College.

### MSC transplantation

For the in vivo MSC homing assays, MSC.AdLuc.LentiR., MSC.AdLuc.E1A, or PBS were injected intravenously into 231-tumor-bearing mice or tumor-free mice (1 × 10^6^ per mouse). For engineered MSC activation, MSC.Adtrack.LentiR. or MSC.Adtrack.E1A were infused into 231-Luc-tumor-bearing mice (1 × 10^6^ per mouse) through tail vein. And for the functional study of CD3-HAC, MSC.CD3-HAC.E1A. or MSC.Adtrack.E1A were administrated intravenously into the mice harboring 231-Luc (1 × 10^6^ per mouse), followed by PBMC infusion 2 days later.

### In vivo bioluminescence imaging

For the in vivo MSC homing assays, we developed an adenoviral vector containing a firefly luciferase reporter gene (Ad-Luc). MDA-MB-231 (231) breast cancer cells (1 × 10^6^ per mouse) were infused intravenously into BALB/c nude mice to establish breast cancer lung metastasis model. Three weeks after MDA-MB-231 cell transplantation, MSC.AdLuc.LentiR., MSC.AdLuc.E1A, or PBS were injected intravenously into tumor-bearing mice or into tumor-free mice (1 × 10^6^ per mouse). Bioluminescence imaging (BLI) was performed using IVIS-Xenogen 100 system (Caliper Lifesciences, USA) at the indicated time points, as previously reported [24, 33]. Quantitative analysis of in vivo luciferase was measured as described Liu et al. [33].

### Ex vivo immunohistochemistry

Lung tissues were harvested from tumor-bearing or tumor-free mice (day 1 for MSC homing assay, day 1, 2, and 5 for engineered MSC activation, day 6 for the study of fusion protein function). Frozen slides (20 μm) were rehydrated in dH_2_O for 5 min, permeabilized in 0.1% Triton X-100 for 10 min, and blocked in 0.1% Triton X-100 with 5% normal donkey serum for 1 h. Samples were stained with primary antibodies (Additional file 1: Table S1) overnight at 4 °C and secondary antibodies (Additional file 1: Table S2) for 2 h at room temperature. Nuclei were stained with DAPI (Sigma). Images were captured by a two-photon laser scanning confocal microscope (OLYMPUS, FV1200 MPE).

### In vivo treatment of breast cancer

MDA-MB-231-Luc (Luc-231) breast cancer cells were infused intravenously into 5–6-week-old female BALB/c nude mice (1 × 10^6^ per mouse). Six days later (day 0), the mice with luciferase signal in the lung were imaged and randomized into five groups as follows: (1) PBS, (2) 5-FU, (3) MSC+PBMC, (4) MSC.CD3-HAC.E1A+PBMC, and (5) MSC.CD3-HAC.E1A+PBMC+5-FU. Hematoxylin and eosin staining was performed to conform the tumor infiltration in the lung. MSCs were co-infected as described above. MSC, MSC.CD3-HAC.E1A, or PBS were injected intravenously into the tumor-bearing mice (day 1, 8, 15; 1 × 10^6^ per mouse) followed by IL-2 pre-activated PBMC administration 3 days later (day 4, 11, 18). The mice were treated with or without 5-FU (i.p., 20 mg/kg) every other day after MSC injection for three times. In vivo luciferase signal was monitored via Xenogen IVIS imaging at indicated time points. For the survival experiment, the endpoint for mice was defined as “found dead” or euthanasia criteria. For the pathology inspection, the brain, spleen, liver, and bone marrow were harvested and stained with hematoxylin and eosin.

### Statistical analysis

Data are represented as mean ± SD. Statistical analysis was performed using GraphPad Prism 6 or Microsoft Excel software. Significance was assayed by an unpaired two-tailed Student *t* test or ANOVA. *P < 0.05; **P < 0.01; ***P < 0.001; ****P < 0.0001.

## Results

### Design and production of CD3-HAC

**Fig. 1:**
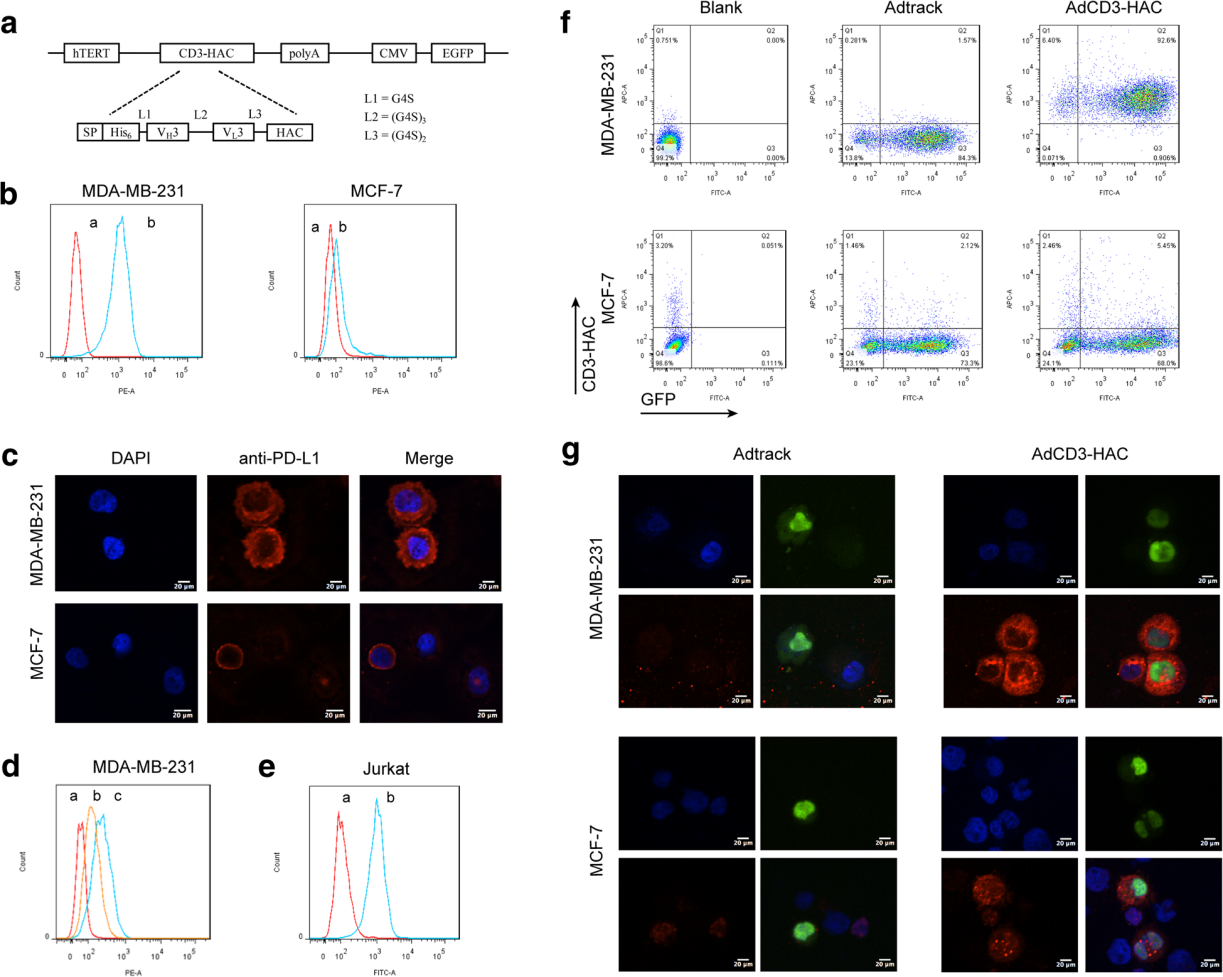
Specific binding capacities of CD3-HAC to PD-L1-positive cells and CD3-positive cells. **a** Schematic representation of adenoviral expression vector for CD3-HAC. hTERT, promoter of human telomerase reverse transcriptase; SP, signal peptide, a murine kappa light-chain leader peptide; His_6_, hexa-histidine tag; G4S, Gly-Gly-Gly-Gly-Ser residues. **b**, **c** PD-L1 expression on breast cancer cells (MDA-MB-231 and MCF-7) detected by flow cytometry and immunofluorescence. (a) Negative control. (b) PE-labeled anti-PD-L1 antibody. Blue (nuclei); red (anti-PD-L1). Scale bar, 10 *μ*m. **d** Competitive binding activity of purified CD3-HAC with anti-PD-L1 antibody on MDA-MB-231 cells. (a) Negative control. (b) CD3-HAC+anti-PD-L1 antibody. (c) Anti-PD-L1 antibody alone. **e** Binding specificity of CD3-HAC to Jurkat cells. (a) Negative control. (b) CD3-HAC. **f**, **g** Flow cytometry and immunofluorescence analyses performed on breast tumor cell lines infected by AdCD3-HAC to detect the binding specificities of CD3-HAC with anti-His antibody. Blue (nuclei). Green (GFP) indicates the cells infected by adenovirus. Red (anti-His for CD3-HAC). Scale bar, 35 *μ* m

To construct this bispecific fusion protein targeting CD3 on T cells and PD-L1 on tumor cells, single-chain variable fragment (scfv) of anti-CD3 antibody was fused to a high-affinity PD-1 sequence (HAC) published by Maute et al. [13], which was a variant of PD-1 ectodomain strongly binding to PD-L1 (Fig. 1a). A hexahistidine-tag (6×His-tag) was added to the amino terminus of the construct to aid in the detection and purification of the products. Then, four adenoviral vectors including AdCD3-HAC, AdCD3scfv, AdHAC, and Adtrack were successfully constructed. The one named AdCD3-HAC was composed of the hTERT promoter fragment, signal peptide, CD3-HAC gene, and the GFP sequence. AdCD3scfv and AdHAC consisted of each binding site of CD3-HAC served as controls for the following function study. The other one named Adtrack containing only the GFP sequence was used as a control vector. We used these four adenoviruses to infect MDA-MB-231 cells or MCF-7 cells at 100 MOI, respectively. To obtain the purified fusion protein, eukaryotic expression vector pcDNA3.1(+) was used with corresponding CD3-HAC gene, which was expressed in adherent 293 T cells. Results from Western blot show the molecular weight of CD3-HAC is about 43.7 kDa (Additional file 1: Figure S3), and the level of CD3-HAC released into the culture was measured by ELISA at the indicated time (Additional file 1: Figure S4).

### CD3-HAC binds specifically both to its target antigens

Firstly, we verified the high expression of PD-L1 on MDA-MB-231 cells (PD-L1^hi^) and low expression of PD-L1 on MCF-7 cells (PD-L1^low^) by flow cytometry and immunofluorescence, respectively (Fig. 1b, c). Then, the purified CD3-HAC was shown to significantly prevent anti-PD-L1 antibody from binding to MDA-MB-231 cells in a competitive binding assay (Fig. 1d). Besides, the purified CD3-HAC could also bind to CD3-positive Jurkat cells via its anti-CD3 scfv domain (Fig. 1e). To further confirm whether the secreted CD3-HAC from tumor cells after being infected by adenovirus could bind to PD-L1 on tumor cells, both flow cytometry and immunofluorescence analysis were performed. As shown in Fig. 1e and f, GFP-positive cells or cells with green fluorescence indicate that cells were infected by adenovirus successfully. For MDA-MB-231 cells, a red fluorescence ring around both infected cells and uninfected cells was shown, which indicated that the localization of CD3-HAC protein was on the cell surface, while weak red fluorescence was observed on PD-L1^low^ MCF-7 cells.

### Specific cytotoxicity against PD-L1^+^ cells

**Fig. 2:**
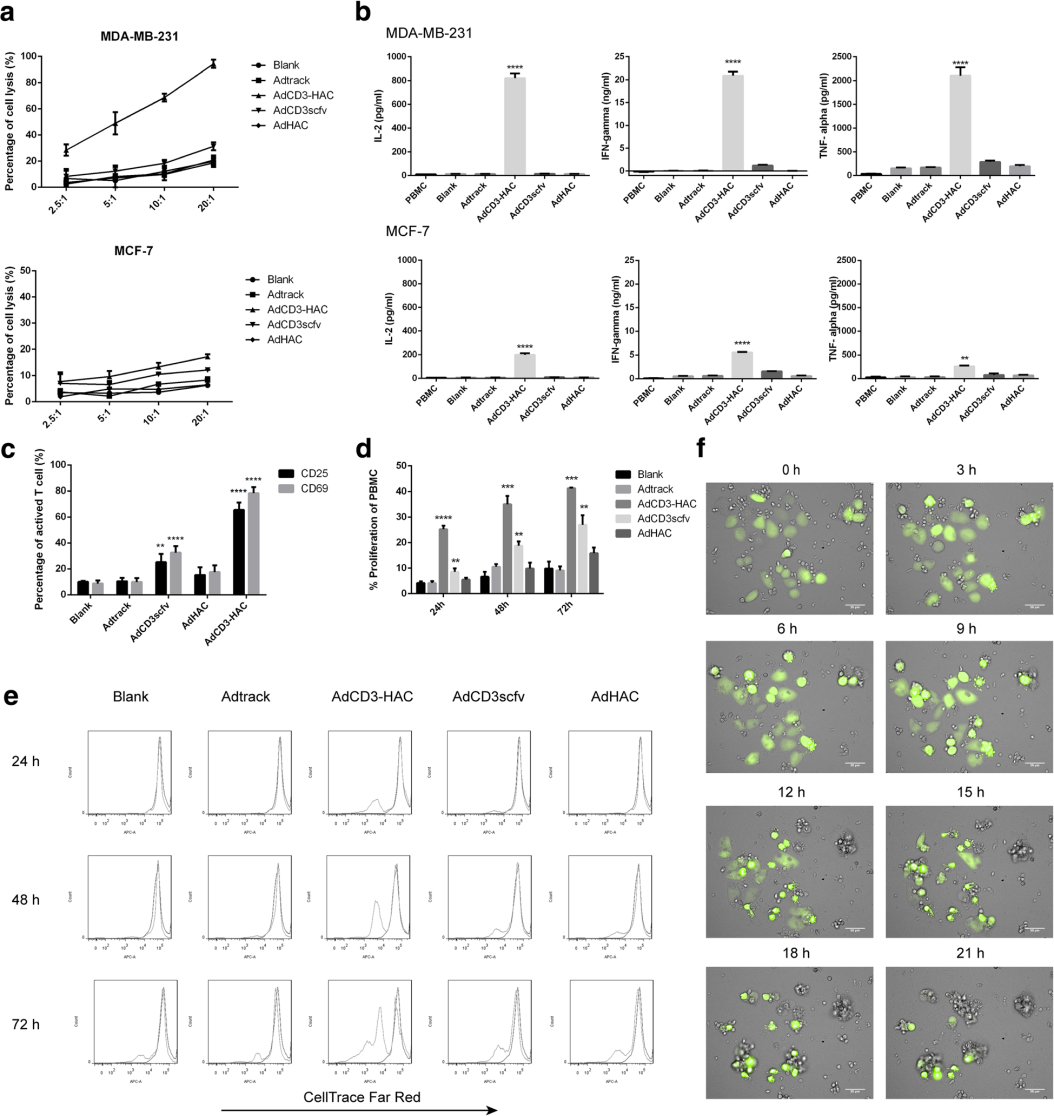
The specific interaction between PBMCs and PD-L1-positive cell lines induced by CD3-HAC. **a** LDH release assays performed on MDA-MB-231 (PD-L1^hi^) cells or MCF-7 (PD-L1^low^) cells infected by various adenovirus in the presence of PBMCs in different effector to target (E:T) ratios for 10 h. **b** Cytokines including IL-2, IFN-γ, and TNF-α in the co-culture supernatants at E:T ratio (10:1) were detected by ELISA kits. **c** Flow cytometry analysis on CD69 and CD25 expression on T cells after co-cultured with adenovirus loaded MDA-MB-231 cells for 24 h. **d** Flow cytometry analysis on the proliferation of PBMCs (labeled with CellTrace™ Far Red) after co-cultured with adenovirus-loaded MDA-MB-231 at E:T radio (10:1) at indicated time. **e** Representative pictures of PBMC proliferation assay. **f** Dynamic observation of cell interaction by living cells workstation. Green-colored cells were AdCD3-HAC-transfected MDA-MB-231, and others were PBMCs. Scale bar, 50 *μ*m. SD shown, *n* ≥ 3. One-way ANOVA with Tukey’s post-test in all cases. ** *P* < 0.01; ***P < 0.001; ****P < 0.0001; compared with Adtrack group

To investigate the specific tumor lysis mediated by CD3-HAC in the presence of T cells, CytoTox96VR Non-Radioactive Cytotoxicity Assay was performed. MDA-MB-231 cells or MCF-7 cells, infected by AdCD3-HAC or control viruses at 100 MOI, were used as targets and co-cultured with PBMCs at different E:T ratios ranging from 20:1 to 2.5:1. As shown in Fig. 2a, MDA-MB-231 cells (PD-L1^hi^) were lysed efficiently at different E:T ratios. And lysis of MDA-MB-231 cells induced by CD3-HAC proceeded in a dose-dependent manner, in which increasing the ratio of E:T resulted in enhanced cytotoxicity. However, for the uninfected or infected by Adtrack cells, PBMCs showed almost no killing function. The intratumor injection of adenoviruses against the MDA-MB-231 cell xenograft tumors was performed to confirm the specific tumor lysis mediated by CD3-HAC (Additional file 1: Figure S8). For the PD-L1^low^ cell line MCF-7, slight cytotoxic death was also detected at the highest E:T ratio, although no statistical significance existed (Fig. 2a). Since the secretion of cytokines served as a main standard to evaluate the activation and cytotoxicity of lymphocytes, the classic cytokines including IL-2, IFN-γ, and TNF-α were measured to reflect the activation of PBMCs. In the supernatant of MDA-MB-231.CD3-HAC+PBMC or MCF-7.CD3-HAC+PBMC co-culture system, the concentrations of IL-2 was 821.70 ± 23.39 pg/mL or 199.60 ± 7.85 pg/mL, IFN-γ was 20.90 ± 0.49 ng/mL or 5.57 ± 0.03 ng/mL, and TNF-α was 2108.00 ± 98.71 pg/mL or 258.40 ± 12.21 pg/mL, respectively, all of which were significantly higher than that of the control groups (Fig. 2b). The expression levels of the early T cell activation marker CD69 and the late activation marker CD25 on CD3-positive cells were also assessed (Fig. 2c). Incubation of PBMCs with MDA-MB-231.CD3-HAC induced a dramatic upregulation of CD69 (78.43 ± 2.54%) and CD25 (65.53 ± 3.28%), while moderate increase was detected of the AdCD3scfv-treated group. It is well known that T cells kill tumors by the perforin/granzyme B pathways. We observed a greater expression of perforin/granzyme B in PBMCs of AdCD3-HAC-infecting group compared to that of control (Additional file 1: Figure S5). Similarly, the percent of PBMC proliferation was substantially higher for AdCD3-HAC-infecting groups as compared to corresponding control (Fig. 2d, e). Moreover, typical cell interaction including binding and then lysis was examined by confocal microscopic video imaging at indicated time points (Fig. 2f).

### Blocking of the PD-1/PDL-1 interaction with HAC increased lymphocyte activation

**Fig. 3:**
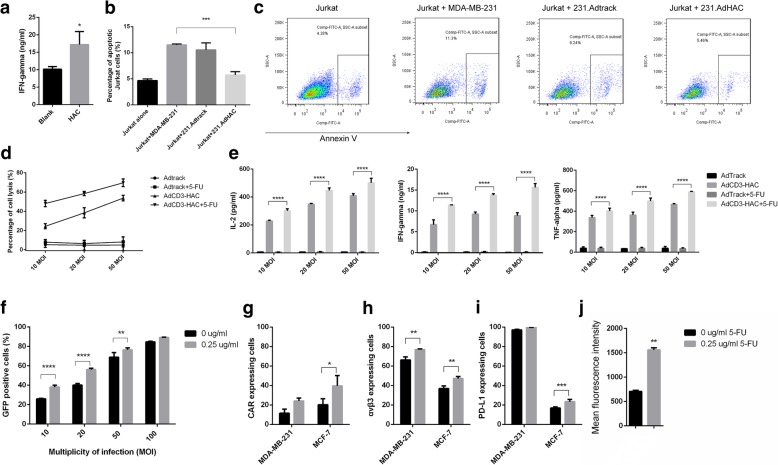
Lymphocytes cytotoxicity mediated by AdCD3-HAC was enhanced by 5-FU through upregulation of CAR and αvβ3. **a** ELISA analyses for IFN-γ on supernatants from 231.CD3scfv cells incubated with PBMCs for 3 days followed by that floating cells were harvested for second-round co-incubation with or without HAC for 5 days. **b** Jurkat T cells were incubated either alone or in co-culture with various virus-loaded MDA-MB-231 cells at the ratio of 1:10 for 10 h. Apoptosis of Jurkat cells was determined by flow cytometry using FITC-Annexin V. **c** Representative images show the percentages of apoptotic Jurkat cells. **d** Cytotoxicity of PBMCs to MDA-MB-231 cells infected with different low MOIs of AdCD3-HAC (E:T = 10:1) with or without 5-FU. **e** Cytokines including IL-2, IFN-γ, and TNF-α in the corresponding co-culture supernatants from **d** were detected by ELISA. **f** Flow cytometry analysis performed on the infection efficiencies of adenovirus to MDA-MB-231 cells, which were pretreated with 5-FU (0, 0.25, and 0.5 μg/mL) for 48 h, at different MOIs. **g**–**i** Flow cytometry analysis on the expression level of CAR, αvβ3, and PD-L1 on the surface of MDA-MB-231 cells and MCF-7 cells treated with low doses of 5-FU for 48 h. **j** Mean fluorescence intensity of PD-L1 on MDA-MB-231 cells treated with 5-FU. SD shown, *n* ≥ 3. Two-tailed unpaired *t* test for **a** and **j** and one-way ANOVA with Tukey’s post-test for **b, e, f, g, h,** and **i**. *P < 0.05; **P < 0.01; ***P < 0.001;****P < 0.0001

In order to determine whether the intervention with high-affinity PD-1 (HAC) could diminish the effects of PD-1/PD-L1 axis in T cells exposed to tumor cells, we set up a two-round co-incubation assay as previously reported [34]. Besides, a 231.CD3scfv cell line, which constitutively expressed anti-CD3 scfv on the surface of MDA-MB-231, was established to induce the directly specific interactions between T cells and target cells [30]. The first-round co-culture with 231.CD3 aimed to upregulate the expression of PD-1 on naive T cells (Additional file 1: Figure S6) leading to tumor cells resistance to CTL-mediated lysis, then IFN-γ secreted from T cells was measured during a second round of exposure to 231.CD3 with or without HAC at low E:T ratio (1:5) [35]. The presence of HAC significantly improved the activation efficacy of PD-1/PD-L1 exhausted T cells (Fig. 3a). Interactions between PD-L1 on tumor cells and PD-1 on T cells are known to lead to anergy or apoptosis of T cells [36]. To further elucidate the PD-1/PD-L1 blockade of HAC, we assessed apoptosis in Jurkat T cells either alone or co-cultured with AdHAC- or Adtrack-loaded MDA-MD-231 cells. As shown in Fig. 3b and c, exposure of Jurkat cells to PD-L1^+^ cells substantially affected their basal level of apoptosis and the presence of HAC notably reversed the apoptosis of Jurkat cells induced by PD-1/PD-L1 axis.

### 5-FU enhanced lymphocyte cytotoxicity mediated by AdCD3-HAC through upregulation of CAR and αvβ3

The adenovirus delivered by gene-modified MSCs was limited, as well as the amount of CD3-HAC concentrated in tumor sites. Now we pursued a combination therapy to improve AdCD3-HAC-mediated lymphocytes cytotoxicity especially at lower MOI. In the previous study, we had demonstrated that pretreatment of 5-FU could promote the lysis of hepatoma cells in the similar system [24, 30]. Here, we discussed the probability of applying 5-FU in the treatment of breast cancer. MDA-MB-231 cells were infected by AdCD3-HAC at 10, 20, or 50 MOI after treatment with low dose of 5-FU (the determination of proper dosage was shown in Additional file 1: Figure S7) and incubated with PBMCs at an E:T ratio of 10:1. AdCD3-HAC group displayed 24.45 ± 2.66%, 38.42 ± 5.28%, and 53.73 ± 2.88% lysis at 10, 20, and 50 MOI, respectively. In contrast, the lysis of MDA-MB-231 cells, which was pretreated with 5-FU, was improved to 48.08 ± 3.35%, 58.46 ± 2.27%, and 69.61 ± 3.98%, respectively (Fig. 3d). Similar results were obtained in assays for cytokine secretion (Fig. 3e). To further investigate the possible mechanism of enhanced lysis triggered by 5-FU, infection efficiency of adenovirus on MDA-MB-231 cells pretreated with 5-FU was measured. As shown in Fig. 3f, dose-dependent increases in viral uptake from 10 to 100 MOI were observed, and dramatic increases in infection efficiency were detected especially at lower MOI. Next, we confirmed that the expression levels of viral attachment receptor CAR and the major internalization receptors αvβ3 were significantly upregulated by the exposure to low dose of 5-FU (Fig. 3g, h). These data indicate that 5-FU sensitizes adenovirus infection and leads to improve cytotoxic effects of PBMCs. Interestingly, pre-treatment with 5-FU substantially increases the expression level of PD-L1 on MCF-7 cells (Fig. 3i), as well as the expression intensity of that on MDA-MB-231 cells (Fig. 3j), which represents the increased binding probability for CD3-HAC.

### Package of adenovirus in E1A-modified MSCs

**Fig. 4:**
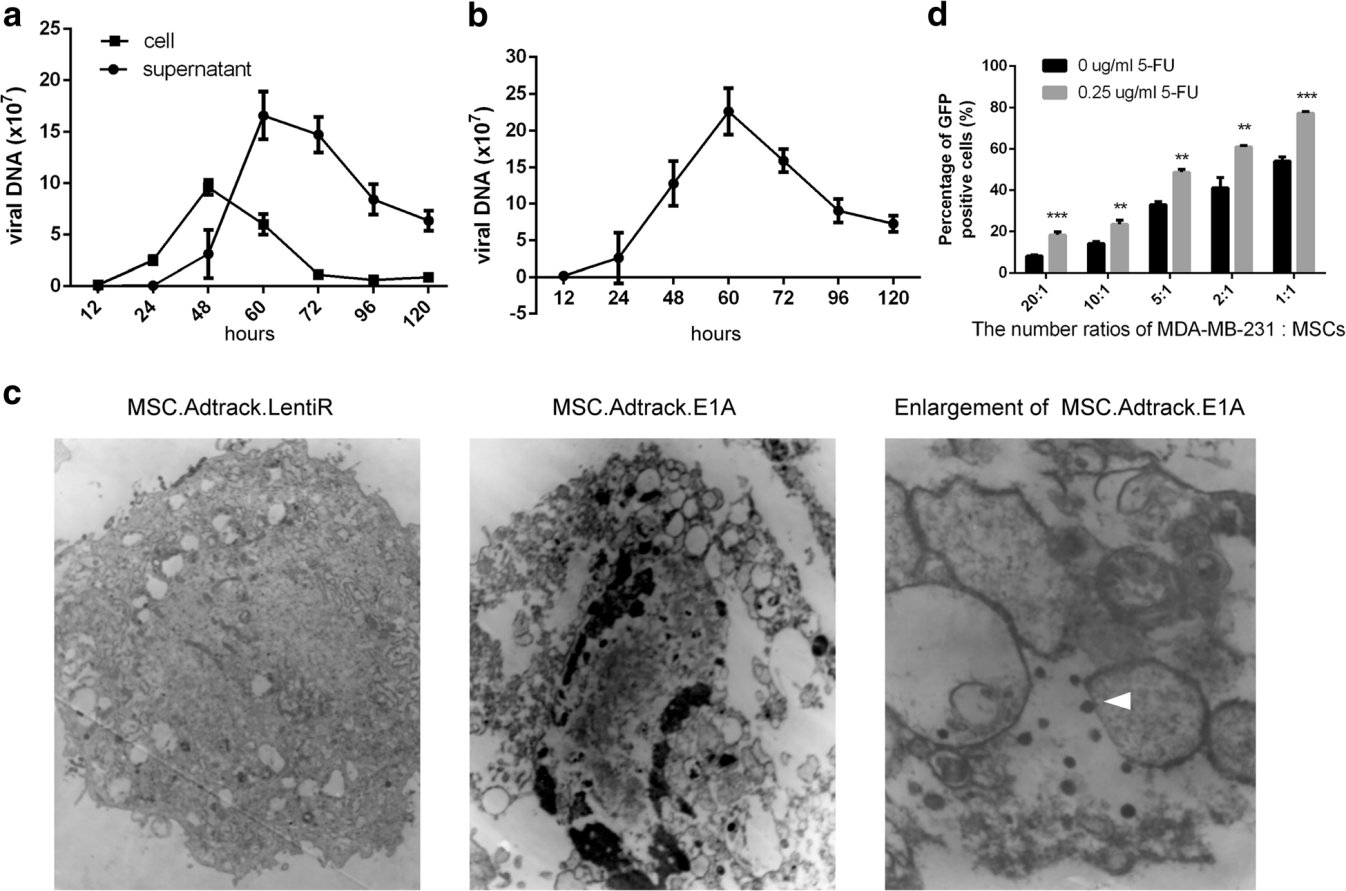
Release of adenovirus by co-infected MSCs and the re-infection ability for tumor cells. **a** Droplet Digital PCR analysis on adenoviral DNA in the intracellular and supernatant from MSC.Adtrack.E1A at indicated time. The infection of LentiR.E1A was set as 0 h. **b** Total DNA numbers of adenovirus (intracellular plus supernatant) in MSC.Adtrack.E1A. **c** Electron micrographs showing viral particles in MSCs 48 h after co-infection. Adenovirus particles were indicated by white arrow. MSC.Adtrack.LentiR. maintained integral cell morphology (left panel); MSC.Adtrack.E1A lysed to release the new packaged adenoviral particles (middle panel); high magnification of MSC.Adtrack.E1A (right panel). **d** Flow cytometry analysis on the re-infection efficiency of new released adenoviruses to MDA-MD-231 cells after co-cultured with different amount of MSC.Adtrack.E1A for 72 h with or without 5-FU. SD shown, *n* ≥ 3. One-way ANOVA with Tukey’s post-test for **d**. ** *P* < 0.01; ***P < 0.001; compared with group in the absence of 5-FU

To ascertain whether E1A-modified MSCs were feasible to serve as adenovirus packaging cells, the exact copy number of hexon gene (late adenoviral gene) in the intracellular and supernatant of co-transfected MSCs (MSC.Adtrack.E1A) was measured by the QX200 Droplet Digital PCR at indicated time. With the increased expression of E1A gradually [24], the adenoviral assembly in intracellular was initiated at 24 h and lasted to 60 h, while the peak level of virus DNA concentration in supernatant delayed to 60 h and remained relatively high level until 120 h (Fig. 4a). The total number of adenoviral DNA (including intracellular and supernatant) replicated in MSC.Adtrack.E1A increased rapidly from 24 h to 60 h, and then declined slowly (Fig. 4b). Furthermore, the intracellular viral particles in MSC.Adtrack.E1A were identified by electron microscopy 48 h after infection (Fig. 4c). To examine the re-infection ability of adenoviruses assembled by E1A-modified MSCs, flow cytometry was applied to measure the infection efficiency of adenoviruses released from MSC.Adtrack.E1A to MDA-MB-231 cells in a co-culture system. The number of GFP-positive cells increased along with the addition of MSCs, ranging from 8.10 ± 0.86% to 54.13 ± 1.28%. Besides, pretreatment with 5-FU significantly improved infection efficiency at all MDA-MB-231:MSCs ratios (Fig. 4d).

### Tropism of MSCs to metastatic breast cancer in vivo

**Fig. 5:**
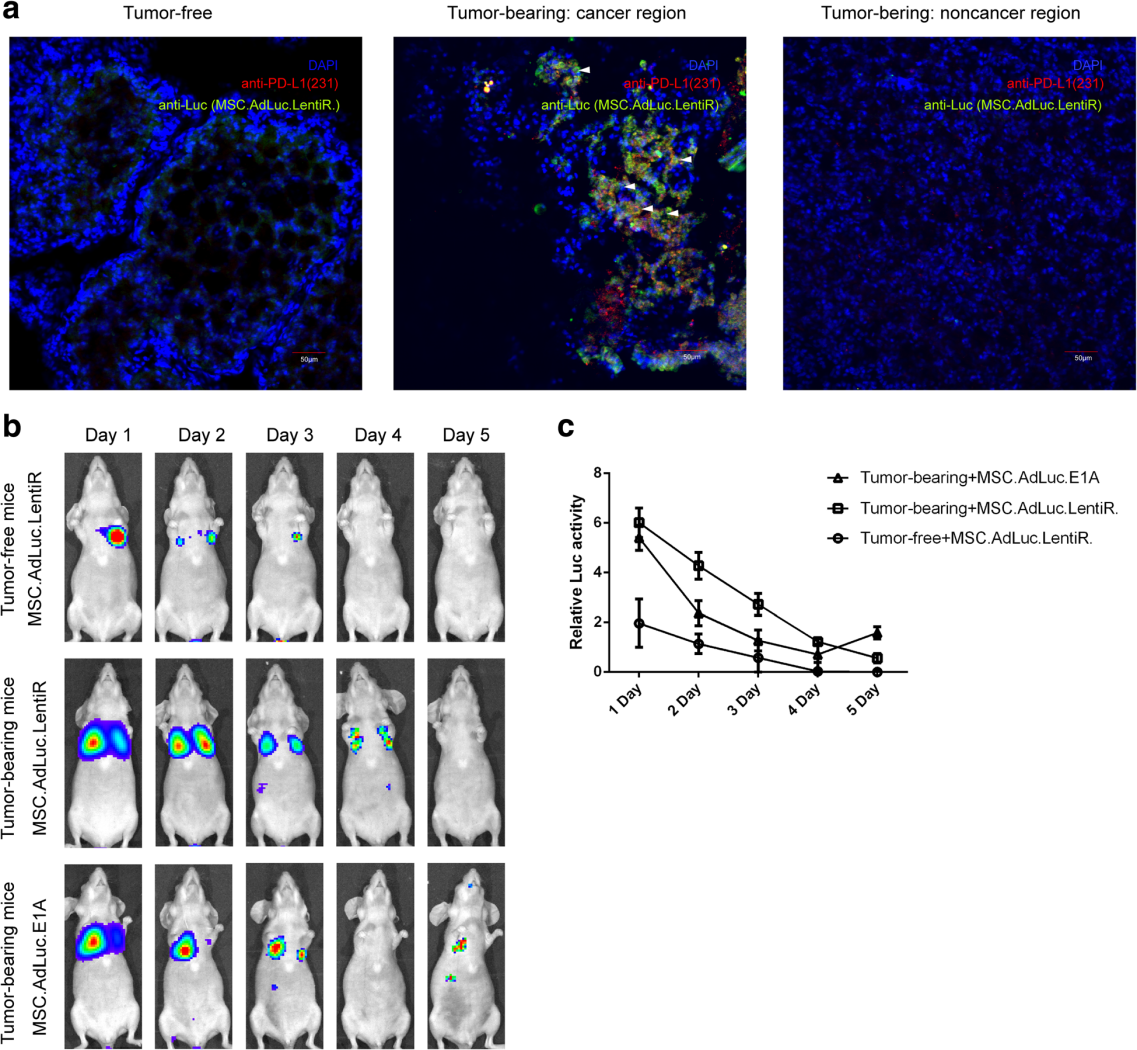
MSCs migrate to metastatic breast cancer in vivo. **a** Frozen sections of the lungs from Luc-231 tumor-bearing and tumor-free mice sacrificed 24 h after MCS.AdLuc.LentiR infusion were stained with anti-PD-L1 (red) for lung metastasis, anti-Luc (green) for luciferase expressed by MSCs, and DAPI (blue). White arrows indicate the co-localization of lung metastatic sites and MSCs. Scale bar, 50 μm. **b** Representative images show the homing capability of different virus-loaded MSCs to tumor sites. MSC.AdLuc.E1A or MSC.AdLuc.LentiR. were intravenously injected into mice hosting MDA-MB-231 in the lung or tumor-free control. Luciferase signal was monitored by bioluminescence imaging using Xenogen imaging system at indicated time. **c** Quantification of luciferase activity of MSCs in the lungs of MDA-MB-231 tumor-bearing or tumor-free mice at different time after MSCs infusion. Relative Luc activity (RLA) = Log_2_ [(luciferase ROI of the mice infused with MSC.AdLuc.E1A or MSC.AdLuc.LentiR.)/(luciferase ROI of control mice infused with PBS)], (*n* = 3)

Wild-type MSCs and gene-modified MSCs were previously suggested to have a homing predisposition to both primary tumor and metastatic niche including breast cancer [29, 32, 33]. To establish a model of micrometastasis in the lung, MDA-MB-231 cells were seeded to BALB/c nude mice via tail vein. Immunohistochemistry was performed to ascertain the tumor foci in the lung (Additional file 1: Figure S10). Here, we first investigated whether co-modified MSCs were able to migrate to tumor sites (Fig. 5a). MSCs labeled with different viruses were infused intravenously into the mice hosting breast cancer cells in the lung or tumor-free control. Ex vivo immunohistochemistry staining demonstrated that co-infected MSCs selectively co-localized with cancer metastasis of tumor-bearing mice (but not tumor-free mice) mice. Next, we examined the specific function of MSC.AdLuc.E1A in vivo, which served as a substitution of MSC.CD3-HAC.E1A and allowed us to monitor the change of MSC vehicle in a visual method (Fig. 5b, c). The intensity of luciferase signal reflects the migratory ability of MSCs to tumor sites. Representative bioluminescent imaging analysis revealed that the systemically administrated MSC.AdLuc.LentiR. can home to and accumulate in tumor sites for up to 3 or 4 days, although part of MSCs were hijacked in the lung [21]. In the MSC.AdLuc.E1A group, intense imaging signal could also be tracked at the tumor sites 1 day after injection, and decreased rapidly from day 2 because of the release of adenoviruses. The signal slightly increased again on day 5, indicating that the recombinant Ad-Luc packaged in MSC.AdLuc.E1A had infected surrounding tumor cells followed with expression of luciferase. In conclusion, these data confirmed that co-injected MSCs specifically migrated to metastatic niche and released adenoviruses at tumor tissues. Additionally, we investigated the duration of gene-modified MSCs in vivo, which provided guidance for the timeline of treatment.

### Delivery of CD3-HAC to metastatic niche through dual-viruses-loaded MSCs in vivo

**Fig. 6:**
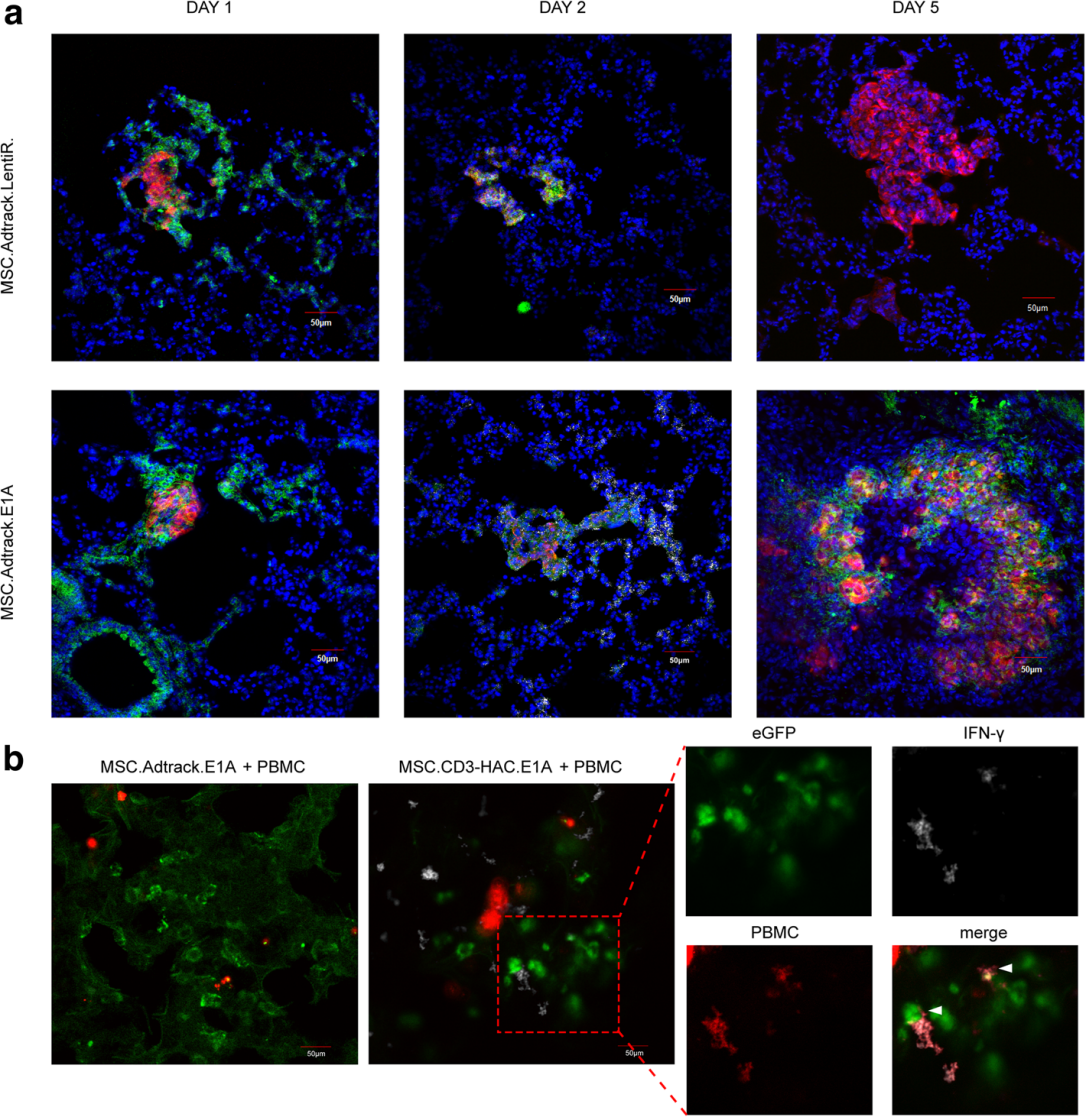
Dual viruses-loaded MSCs delivering CD3-HAC to metastatic niche. **a** Frozen sections of the lungs from 231-Luc tumor-bearing mice sacrificed at indicted time points after MSC.Adtrack.LentiR. or MSC.Adtrack.E1A infusion showed the change of gene expression of engineered MSC in vivo. Blue (nuclei). Green (GFP) represented the cells infected by adenovirus. Red (anti-Luc) represented MDA-MB-231 cells. White (DsRed) represented the expression of E1A. Pictures of each channel were displayed in Additional file 1: Fig. S9. **b** MSC.CD3-HAC.E1A or MSC.Adtrack.E1A were injected intravenously into 231-Luc hosting mice, followed by PBMCs infusion after 2 days. IFN-γ^+^ cells were detected in tumor sites. White arrows indicated the interaction between the IFN-γ^+^ cells and adenovirus-infected tumor cells. Green (GFP) represented the cells infected by adenovirus. Red (Cell Trace™ Far Red) represented PBMCs. White (anti-IFN-γ). Scar bar, 50 μm

To further investigate the “two-step” gene expression of dual-viruses-transfected MSCs in vivo, we used ex vivo immunohistochemistry (IHC) in 231-Luc tumor-bearing mice (Fig. 6a). Results from immunofluorescence staining demonstrated that GFP-positive cells migrated towards metastasis through vessels in MSC.Adtrack.E1A and mock MSCs groups 1 day after MSC administration. We speculated that they were the virus-loaded MSCs homing to the tumor sites. On the second day, MSCs continuously expressed GFP and co-localized with tumor cells in the lung. The reporter gene DsRed observed in the MSC.Adtrack.E1A group indicated that co-expressing E1A gene started to induce the adenoviral replication in MSCs, which was the first step of this gene delivery system. On day 5, some GFP-positive cells were detected in the MSC.Adtrack.E1A group, instead of in the E1A-deficient group. We speculated that these phenomena were induced by the re-infection of the adenoviruses released from those homed MSC.Adtrack.E1A, which was the second step of transgene delivery. Next, we confirmed the specific effect of CD3-HAC transported by MSC.CD3-HAC.E1A in vivo (Fig. 6b). Forty-eight hours after PBMCs systemically transplanted, lymphocytes labeled with Cell Trace™ Far Red Fluorescence were observed in tumor sites in both groups. Since the fluorescence intensity of Cell Trace™ Far Red decreased with lymphocytes activation and proliferation, for convenience of observation, we focused on interesting fields under high magnification. IFN-gamma-positive cells, which indicated the lymphocytes activated by fusion protein CD3-HAC, were observed in the MSC.CD3-HAC.E1A group. Notably, typical cell interaction between lymphocytes and tumor cells was also detected, and GFP-positive cells were remarkably lysed compared to the mock MSC group. Collectively, these data suggest that gene-modified MSCs selectively migrate to the metastatic niche followed by releasing adenoviruses which could infect tumor cells, in which CD3-HAC was specifically secreted to trigger T cells to kill tumor cells.

### Antitumor potential of MSC.CD3-HAC.E1A in combination with 5-FU against MDA-MB-231 xenograft tumors

**Fig. 7:**
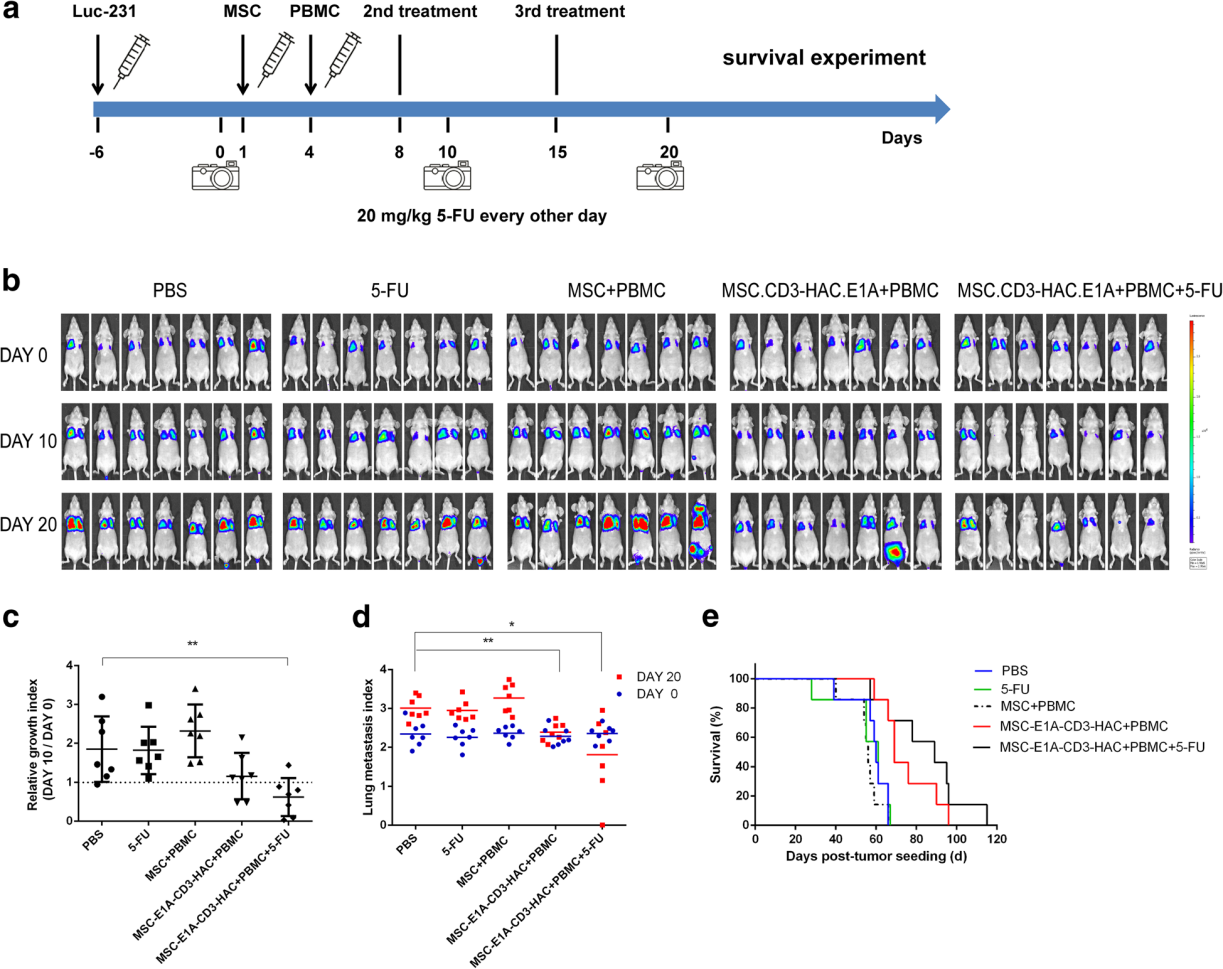
Tumor suppression of MSC.CD3-HAC.E1A in combination with 5-FU against MDA-MB-231 lung metastasis. **a** Design and timeline for tumor therapy. BALB/c nude mice were injected with MDA-MB-231-Luc cells (Luc-231, 1 × 10^6^ per mouse) via tail vein. Seven days later (day 1), MSCs (1 × 10^6^ per mouse) were administrated intravenously into the Luc-231 tumor-bearing mice. Then, mice received intravenous injection of PBMCs (5 × 10^6^ per mouse) on day 4. Besides, 5-FU was given (i.p., 20 mg/kg) every other day along the treatment. The same treatment was performed another two times as indicated. i.p., intraperitoneal. **b** IVIS imaging of MDA-MB-231-Luc cells were shown for all groups. IVIS imaging was taken before (day 0) and after MSCs treatment (day 10 and day 20). Quantification of luciferase signals in the lung on day 10 (**c**) and day 20 (**d**). **e** Mouse survival after MSC.CD3-HAC.E1A treatment (*n* = 7 per group). In **c**, relative growth index = luciferase read on day 10 /luciferase read on day 0. In **d**, lung metastasis index = log10 [(luciferase read of the tested mice)/(luciferase read of average for tumor-free mice)]. *P <0.05 and **P <0.01 compared with the PBS group. In **e**, *P* = 0.0108, MSC.CD3-HAC.E1A+PBMC versus PBS; *P* = 0.0161, MSC.CD3-HAC.E1A+PBMC+5-FU versus PBS. Median survival (days): PBS, 60; 5-FU, 61; MSC+PBMC, 56; MSC.CD3-HAC.E1A+PBMC, 69; MSC.CD3-HAC.E1A+PBMC+ 5-FU, 89

To evaluate the efficacy of engineered MSC infusion system for micrometastasis, a 231-Luc lung metastatic model in BALB/c nude mice was established, in which virus-loaded MSCs and PBMCs were injected intravenously, followed by 5-FU. MSCs or virus-loaded MSCs were injected 2 days before PBMC infusion to allow time for colocalization with tumors and replication of adenovirus (Fig. 7a). Dosing interval of the MSC administration was conformed via the typical persistent period of MSCs in the tumor sites discussed above [33]. The mice were imaged by bioluminescent IVIS imaging to monitor the tumor burden within the lungs every 10 days (Fig. 7b). To quantitatively analyze the tumor growth, we refer to the methods provided in previous study [33]. Compared to the initial intensity before treatment, luciferase signals were decreased in mice treated with MSC.CD3-HAC.E1A combined with PBMCs and 5-FU (Fig. 7c). Additionally, mice handled with MSC.CD3-HAC.E1A and PBMCs almost maintained no growth (Fig. 7d). However, mice in the PBS or 5-FU control group showed increase in tumor mass over time as tumor continued to grow. Notably, the unmodified MSC systemic administration slightly promoted the growth of tumor, which was consistent with previous reports [37] and indicated that MSCs should be used with cautions in clinical applications. After the first treatment (day 10), luciferase signals in mice treated with MSC.CD3-HAC.E1A and PBMCs showed no statistically significant decrease compared with the control groups. Since 5-FU was applied to improve the utilization of adenovirus and induce the expression of PD-L1, the 5-FU-added group displayed tumor regression compared to PBS or 5-FU group (Fig. 7c). On day 20, the MSC.CD3-HAC.E1A+PBMC group began to show evident antitumor effect compared to the control groups (Fig. 7d), while the MSC.CD3-HAC.E1A+PBMC+5-FU group maintained a lower tumor burden. Additionally, the survival time of mice was also significantly prolonged after transplanted with MSC.CD3-HAC.E1A and PBMCs compared to the PBS or 5-FU group (Fig. 7e). Potential tissue damages in other organs were also evaluated to ascertain the safety of this therapeutic strategy. The bone marrow, liver, brain, and spleen were particularly concerned [38] and stained by hematoxylin and eosin. No obvious damages were detected except for a handful of tumor infiltration (Additional file 1: Figure S11). These data indicated that modified MSCs serving as delivery vehicles do not induce side effects.

## Discussion

Here, we sought to explore whether it might be possible to produce curative outcomes among mice harboring lung metastasis of breast cancer through local release of a bispecific fusion protein in the presence of 5-FU or not. We showed specifically expressed CD3-HAC protein by tumor cells in a controlled spatiotemporal manner using a proved MSC.E1A delivery system conferring certain efficacy to trigger lymphocytes and bridged T cells to PD-L1-expressing tumor cells. And the antitumor effect of this targeted therapeutic system in combination with 5-FU was confirmed in vivo.

Although with decades of exploration, little progress was achieved in the treatment of metastasis. Several studies described TNBC as the most immunogenic subtype with increased tumor infiltrating lymphocytes (TILs) and PD-L1 expression compared to other breast cancers [39, 40], leading to various clinical trials of PD-1/PD-L1 blockade therapy. Although preliminary, the overall response rate of monotherapy with anti-PD-1/PD-L1 therapy in metastatic TNBC ranged from 4 to 20% [41]. The immunologic inertia of metastatic lesions [42] and immune privilege mechanism in cold tumor cases [43] count for potential explanations of the low response rate in TNBC, both highlight the critical role of addressing deficient T cell priming in PD-1/PD-L1-refractory cancers. Harnessing and recruitment of T cells via bispecific proteins serves as an effective approach to prime immune response, which bind one arm to T cell activation domain and bind the other arm to a tumor-associated antigen on the target cell [44]. Here, we constructed CD3-HAC composed by anti-human CD3scfv sequence and a high affinity PD-1 sequence (HAC) in adenoviral vector. To enhance the anti-tumor specificity and reduce the potential risk to normal cells, we employed hTERT promoter to operate the expression of CD3-HAC fusion protein. The surface binding to cancer cells of CD3-HAC was examined by immunofluorescence and flow cytometry analysis on PD-L1^hi^ and PD-L1^low^ cell lines (Fig. 1f). CytoTox96VR Non-Radioactive Cytotoxicity Assay claimed that the lysis of tumor cells induced by CD3-HAC was significantly proportional to the amount of PD-L1 expressed by the target cells within 10 h (Fig. 2a). Moreover, the function of CD3-HAC was comprehensively tested from different aspects, including the expression of activation markers on lymphocytes by flow cytometry, cytokine levels by ELISA, and typical cell interaction by confocal microscopic video imaging.

Besides the tumor cells, various host immune cells and stromal cells in the complex tumor microenvironment maintain the expression of PD-L1, particularly myeloid cells and tumor-associated macrophages, IFN-γ dependent or not [45, 46]. Since CD3-HAC targets PD-L1-positive cells, specious “on target/off tumor” effect was reasoned. Recently, it suggested that PD-L1 expression in both the host and tumor compartment contributes to immune suppression in a non-redundant way and both predicted the sensitivity of therapeutic agents targeting the PD-L1/PD-1 axis [45]. We inferred that blockage of host-derived PD-L1 and inducing lysis of immunosuppressive cells by CD3-HAC played essential role in damaging tumor microenvironment and reversing immune tolerance.

To concentrate the immune response strictly within the breast cancer metastases and avoid the potential adverse effects of immunomodulator, we hypothesized that an adenovirus-loaded MSC.E1A can be used for such approach. MSCs are multi-potent cells derived from multiple tissues [47], which have been proven safe for clinical applications in the treatment of various diseases [22]. In previous study, we have demonstrated that gene-modified MSCs could maintain their properties after virus infection, including their surface markers, tri-lineage differentiation, proliferation, and the capacity to migrate towards tumor cells both in vitro and in vivo experiments [44]. And several previous investigations in our laboratory have proved this MSC-based delivery strategy in subcutaneous and orthotopic hepatocarcinoma xenograft model [29, 30, 32]. In the present study, the efficient delivery of therapeutics to metastases was concerned, which is the crucial superiority of MSC.E1A over other intratumoral methods, especially for minimal lesions. And the tropism of MSCs towards metastasis in mice bearing with MDA-MB-231 cells was confirmed in in vivo imaging system. Furthermore, the infection ability and following function of new generated AdCD3-HAC were demonstrated by ex vivo immunohistochemistry (Fig. 6b).

The appropriate expression of E1A is the most essential element in determining the spatiotemporal fate of MSC.E1A transfer system, since the complement of E1A gene in host cells will concomitantly switch on the viral replication of adenovirus serotypes 5 (Ad5) [48]. To avoid the leakage of adenovirus before MSCs located in tumor sites, tissue-specific promoter could be utilized to regulate E1A gene involved in MSC differentiation cascades [29, 33]. However, given the absence of proper promoters for most cancer types, adenovirus-loaded MSC.E1A taking advantage of the delay of lentiviral gene expression to limit the E1A activation could be explored with broad adaptability [24, 30]. Adenoviral replication was observed in MSCs 24 h after LentiR.E1A infection and reached the peak at 48 h, followed with the release of adenoviral particles into supernatant (Fig. 4a). Furthermore, the luciferase bioluminescence and immunofluorescence were performed to investigate the kinetic distribution of systemically administered MSCs (Fig. 5), which confirmed the preferential localization of MSCs to tumor sites within 12 h [33]. Based on these set of data, there is sufficient time for virus-loaded MSCs to arrive at metastatic niche before lysis by adenoviral production. Although it was unfeasible to guarantee the migration of all MSCs, we confirmed that no significant damage was detected in the bone marrow, liver, or brain tissue as a result of systemic treatment with MSC.E1A (Additional file 1: Figure S11).

Note that MSCs have previously been suggested to regulate cancer progression in various researches, both positively and negatively [47, 49, 50]. In this study, MSCs themselves were observed to slightly promote the growth of tumor, other than the adenovirus-loaded MSC.E1A. In previous research, we found that MSCs contribute to the immunosuppressive microenvironment via several pathways in subcutaneous Raji xenografts [44]. However, we do not consider it as a major issue in this system, since the adenovirus-loaded MSC.E1A only stayed in tumors for less than 4 days (Fig. 5b) and have been proved to be disrupted both in vitro and in metastatic niche due to the adenovirus production (Figs. 4c and 6a), which avoided the potential immunosuppression. This suggested that adenovirus-loaded MSC.E1A can be an efficient and safe delivery system against cancer, compared to non-self-destructing MSCs.

Additionally, we combined low dose of 5-FU with this local immunotherapy and intended to increase the utilization of new generated AdCD3-HAC in metastasis and promote antitumor effect. Consistent with previous researches, the expression of CAR and avβ3 on breast cancer cells, essential for adenovirus internalization, were upregulated in the presence of 5-FU. The infection efficiency of adenovirus was consequently increased than that of adenoviral treatment alone, particularly at the lower MOI, and led to improved cytotoxic effect of PBMCs. In established breast cancer xenograft, tumor suppression and survival of the mice was significantly enhanced in the group of MSC.E1A.CD3-HAC+PBMC+5-FU.

Finally, we demonstrated this strategy in shifting the PD-L1-mediated immune evasion mechanism to an efficient therapeutic target. The identification of tumor-associated antigens (TAAs) is at the core of immunotherapeutic interventions; however, available TAAs are limited in the treatment of solid tumor due to the high heterogeneity of malignancy and selective pressure of immune system. PD-L1 is a “broad spectrum” tumor biomarker constructively expressed or responded to interferons (IFNs) [51]. Bispecific CD3-HAC expectedly induced the cytotoxic T lacking PD-L1, although the killing effect remains modest within the first 10 h of interaction (Fig. 2a), the concentrated CD3-HAC protein can boost the tumor infiltrating lymphocyte primarily, and consequently increase the expression of PD-L1 on cancer cells driven by IFN-γ, which represents a reinforced positive feedback loop in the long run. Moreover, the enhanced expression of PD-L1 was accidentally observed by the 5-FU pretreatment (Fig. 3i), which was consistent with previous reports that PD-L1 expression in tumor cells was considerably increased after chemotherapy [52, 53]. We confirmed that the upregulated PD-L1 on tumor cells provided more potential binding sites for CD3-HAC combined with the increased uptake of adenoviruses leading to improved cytotoxic effect of PBMCs (Fig. 3d). Both the innate or adaptive immune resistance can be exploited to overcome the antigen limitations by this CD3-HAC locally released system and achieve cascade-like treatment at the tumor site. Therefore, the CD3-HAC bispecific fusion protein could be served as an effective approach to enhance immune response in tumor microenvironment with different immune phenotypes.

## Conclusions

We have focused on lung metastasis of breast cancer to highlight a spatiotemporally defined manner and antitumor effects of T cells induced by CD3-HAC secreted from tumor cells in the aid of MSC.E1A delivery system towards micrometastasis. Notably, this MSC.E1A-based strategy could be popularized to resolve the problem of metastasis of other cancers and might be a potential cellar platform for the transfer of various immuno-oncology drugs or genetic engineering drugs.

## Additional file

Additional file 1: **Figure S1.** Schematic representation of the therapeutic strategy. **Figure S2.** Schematic representation of lentiviral expression vector for E1A. **Figure S3.** Western blot analysis of CD3-HAC. **Figure S4.** Transduced MDA-MB-231 cells secreted CD3-HAC constantly. **Figure S5.** The expression of Perforin and Granzyme B of PBMC was measured in co-culture system. **Figure S6.** The expression of PD-1 on CD8^+^ cells was measured by flow cytometry after co-incubation with 231.CD3 for 3 days. **Figure S7.** The inhibitory curve of MDA-MB-231 cells treated with 5-FU. **Figure S8.** Intratumor injection of adenoviruses against the MDA-MB-231 cells xenograft tumors. **Figure S9.** Each fluorescence channel in Fig. 6a. **Figure S10.** Immunohistochemical analysis of the lung. **Figure S11.** Dual viruses infected MCSs causing no detectable side effects in vivo. **Table S1.** Primary antibodies. **Table S2.** Secondary antibodies. (DOCX 129760 kb)

## Abbreviations

BiTE: Bispecific T cell engager
BLI: Bioluminescent imaging
CAR: Chimeric antigen receptor
ELISA: Enzyme-linked immune sorbent assay
HAC: High-affinity consensus
hTERTp: Human telomerase reverse transcriptase promoter
Luc: Luciferase
MOI: Multiplicity of infection
MSCs: Mesenchymal stromal cells
PBMCs: Peripheral blood mononuclear cells
PBS: Phosphate-buffered saline solution
PCR: Polymerase chain reaction
scfv: Single-chain variable fragment
