# Supplementary material for: Bispecific CD3-HAC carried by E1A-engineered mesenchymal stromal cells against metastatic breast cancer by blocking PD-L1 and activating T cells
Source: J Hematol Oncol. 2019 Apr 25;12:46. doi: 10.1186/s13045-019-0723-8 (PMC6482514; doi:10.1186/s13045-019-0723-8)
Supplement: Supplementary file 1 — Figure S1. Schematic representation of the therapeutic strategy. Figure S2. Schematic representation of lentiviral expression vector for E1A. Figure S3. Western blot analysis of CD3-HAC. Figure S4. Transduced MDA-MB-231 cells secreted CD3-HAC constantly. Figure S5. The expression of Perforin and Granzyme B of PBMC was measured in co-culture system. Figure S6. The expression of PD-1 on CD8+ cells was measured by flow cytometry after co-incubation with 231.CD3 for 3 days. Figure S7. The inhibitory curve of MDA-MB-231 cells treated with 5-FU. Figure S8. Intratumor injection of adenoviruses against the MDA-MB-231 cells xenograft tumors. Figure S9. Each fluorescence channel in Fig. 6a. Figure S10. Immunohistochemical analysis of the lung. Figure S11. Dual viruses infected MCSs causing no detectable side effects in vivo. Table S1. Primary antibodies. Table S2. Secondary antibodies. (DOCX 129760 kb) [file 13045_2019_723_MOESM1_ESM.docx]

**Supplementary Materials for**

**Bispecific CD3-HAC carried by E1A-engineered mesenchymal stromal cells against metastatic breast cancer by blocking PD-L1 and activating T cells**

**Supplementary Figures**

**Fig. S1 Schematic representation of the therapeutic strategy.**

**Fig. S2 Schematic representation of lentiviral expression vector for E1A.**

**Fig. S3 Western blot analysis of CD3-HAC.**

(**a**) The supernatants of 293T cells after transfection with pcDNA3.1(+)-CD3-HAC were harvested and assayed for CD3-HAC using anti-His tag antibody. (**b**) Western blot analysis on the purified protein. (**c**) MDA-MB-231 cells or MCF-7 cells were infected with different adenoviruses, and the expression of proteins in cells were detected by western blot analysis with anti-His tag antibody. AntiCD3scfv and HAC served as controls.

**Fig. S4 Transduced MDA-MB-231 cells secreted CD3-HAC constantly.**

MDA-MB-231 cells were infected by AdCD3-HAC at 100 MOI and the level of CD3-HAC released into culture was measured by ELISA at the indicated time.

**Fig. S5 The expression of Perforin and Granzyme B of PBMC was measured in co-culture system.**

**Fig. S6 The expression of PD-1 on CD8^+^ cells was measured by flow cytometry after co-incubation with 231.CD3 for 3 days.**

**Fig. S7 The inhibitory curve of MDA-MB-231 cells treated with 5-FU.**

**Fig. S8 Intratumor injection of adenoviruses against the MDA-MB-231 cells xenograft tumors.**

Tumor cells (5×10^6^) in 100 μL PBS were injected into the mammary fat pad in BALB/c nude mice (female, 5–6 weeks of age; PUMC, China) 1 day after the application of total body irradiation (300 cGy). When tumor size reached 60-80 mm^3^, the mice were treated with Adtrack (6.4×10^7^ pfu /50μL per mouse) or AdCD3-HAC (6.4×10^7^ pfu /50μL per mouse) by means of intratumoral injection, followed by PBMCs (6×10^6^) infusion one day later.

**Fig.S9 Each fluorescence channel in Fig. 6a**. DAPI (blue), 231-Luc (red), eGFP (green), DsRed (white). Scale bar, 50 μm.

**Fig. S10 Immunohistochemical analysis of lung.**

Tumor metastasis in lung were observed by H&E staining one week after intravenously injection of MDA-MB-231 cells into mice. Scale bar, 100 μm.

**Fig. S11 Dual viruses infected MCSs causing no detectable side effects in vivo.**

Representative images of sectioned organs with H&E staining indicated no detectable tissue damages in liver, spleen, brain and bone marrow of tumor-bearing mice treated with MSC.CD3-HAC.E1A+PBMC or MSC.CD3-HAC.E1A+PBMC+5-FU. Scale bar, 100 μm.

**Table S1. Primary antibodies.**

| **Antigen** | **Company** | **Catalog #** | **Dilution** | **Notes** |
| --- | --- | --- | --- | --- |
| PD-L1 | Abcam | ab213524 | 1: 100 | Fig.5a |
| eGFP | Abcam | ab183734 | 1: 100 | Fig.5a & 6 & S8 |
| PD-L1 | R&D | MAB1561 | 1: 100 | Fig.1c |
| 6×His tag | MBL | D291-3 | 1: 1000 | Fig.1f,g |
| 6×His tag | MBL | D291-A48 | 1: 1000 | Fig.1e |
| Firefly luciferase | Abcam | ab185924 | 1: 100 | Fig.6a & S8 |
| IFN-gamma | Abcam | ab133566 | 1: 100 | Fig.6b |
| CAR | Bioss | bs2389R | 1: 100 | Fig.3g |

**Table S2. Secondary antibodies.**

| **Tag** | **Company** | **Species** | **Catalog #** | **Dilution** |
| --- | --- | --- | --- | --- |
| PE | BD | Rat α mouse | 55370 | 1: 500 |
| Alexa Fluor 647 | CST | Rat α mouse | 4410 | 1: 500 |
| Alexa Fluor 647 | Abcam | Donkey α rabbit | ab150075 | 1: 500 |
| Alexa Fluor 568 | Abcam | Goat α rabbit | ab175471 | 1: 500 |
